# Changes in physical activity after joining a bikeshare program: a cohort of new bikeshare users

**DOI:** 10.1186/s12966-022-01353-6

**Published:** 2022-10-04

**Authors:** Amy H. Auchincloss, Yvonne L. Michael, Saima Niamatullah, Siyu Li, Steven J. Melly, Meagan L. Pharis, Daniel Fuller

**Affiliations:** 1 Dornsife School of Public Health, Drexel University, 3215 Market Street, Philadelphia, PA 19104 USA; 2 Urban Health Collaborative, Drexel University, Philadelphia, PA USA; 3 Philadelphia Department of Public Health, Philadelphia, PA USA; 4 School of Human Kinetics and Recreation, Memorial University of Newfoundland, St. John’s, NL Canada

**Keywords:** Physical activity, Active transportation, Built environment, Cohort studies, Bicycling, Cycling

## Abstract

**Background:**

There are hundreds of bikeshare programs worldwide, yet few health-related evaluations have been conducted. We enrolled a cohort of new bikeshare members in Philadelphia (Pennsylvania, USA) to assess whether within-person moderate and vigorous physical activity (MVPA) increased with higher use of the program and whether effects differed for vulnerable sub-groups.

**Methods:**

During 2015–2018, 1031 new members completed baseline and one-year follow-up online surveys regarding their personal characteristics and past 7-day MVPA minutes per week (minutes per week with- and without walking). Participants were linked to their bikeshare trips to objectively assess program use. Negative binomial (for continuous outcomes) and multinomial (for categorical outcomes) regression adjusted for person characteristics (socio-demographics, health), weather, biking-infrastructure, and baseline biking.

**Results:**

Participant median age was 30, 25% were of Black or Latino race/ethnicity, and 30% were socioeconomically disadvantaged. By follow-up, personal bike ownership increased and 75% used bikeshare, although most used it infrequently. Per 10 day change in past year (PY) bikeshare use, non-walking MVPA min/wk increased 3% (roughly 6 min/wk, *P* < 0.014). More days of bikeshare was also associated with change from inactive to more active (odds ratio for ≥ 15 days in PY vs. no bikeshare use 1.80, CI 1.05–3.09, *P* < 0.03). Results were consistent across vulnerable sub-groups. In general, impacts on MVPA were similar when exposure was personal bike or bikeshare.

**Conclusions:**

Bikeshare facilitated increases in cycling, slightly increased non-walking MVPA, and showed potential for activating inactive adults; however, for larger program impact, members will need to use it more frequently.

**Supplementary Information:**

The online version contains supplementary material available at 10.1186/s12966-022-01353-6.

## Introduction

Physical activity has been popularly referred to as the best medicine due to its beneficial effects on a wide range of health outcomes [1, 2] yet upwards of 20% of US adults are inactive [3] and more than 50% do not attain sufficient levels of aerobic physical activity [4]. Use of active modes of transportation (walking and biking) have been associated with higher overall levels of physical activity [5]. Bicycle sharing programs could play an important role in promoting active transportation and increase levels of physical activity [6, 7].

More than 1000 bikeshare programs exist worldwide [8], yet few health-related evaluations exist of these programs. Existing studies have documented increases in cycling behaviors in cities with bikeshare, with much of the evidence coming from the International Bikeshare Impacts on Cycling and Collisions Study [9]. For example, Hosford et al. [10], used three years (2012–2014) of the study’s repeated-cross-sectional data from two cities with recently implemented programs (Chicago and New York) and three cities without a bikeshare program at the time of the study (Vancouver, Detroit, Philadelphia). The study found that past-week cycling at follow-up was higher among residents living in cities with newly implemented bikeshare programs over the follow-up period (relative to the baseline period and to residents who did not live within the program’s target area or did not live in a city with bikeshare). Single-city studies found similar results [6, 11].

Repeated cross sectional studies have provided evidence of population increases in cycling attributable to bikeshare [6, 10–12]. However, no evaluations have assessed within-person changes in overall physical activity in response to joining bikeshare, leaving unanswered some key questions related to the health enhancing potential of these interventions [7]. Increased cycling resulting from bikeshare use could lead to reductions in other moderate-vigorous activity, essentially a replacement of one activity for another, leading to no net increase in overall activity or potentially a decrease in overall activity [13]. Alternatively, bikeshare use could be a gateway activity in which uptake in cycling occurs on top of other forms of existing activity or leads to new forms of activity outside of bikeshare use, as users become more confident of their ability to engage in physical activity [14].

Cities have supported bikeshare in large part because of its role in alleviating transit congestion and extending the transportation network to areas that are not well-serviced by bus/rail [15]. However, bikeshare programs have been criticized for their inattention to equity issues [16, 17]. Lower income and minority populations tend to be disproportionately transit-dependent yet have less bikeshare station access and may lack the means to pay for membership (due to cost and requirements that members have a credit card for billing and bicycle-deposit) [17–19]. Data from bikeshare programs have confirmed that bikeshare members tend to be younger, Whiter, better educated and wealthier than residents of the city’s bikeshare target area [18, 20, 21]. Given baseline inequities, bikeshares may exacerbate health inequalities [7, 22]. No study has specifically examined this question.

In spring 2015, the City of Philadelphia (USA), launched a bicycle sharing program called Indego [23]. The program has grown to a service area of approximately 20 square kilometers (8 square miles), with over 1500 bicycles docked at over 165 stations [24]. Philadelphia has the highest poverty rate among the top 10 largest U.S. cities and was one of the first cities to design an equity-focused bikeshare program [25]. The City implemented various strategies to meet equity objectives, for example: placing stations in low income areas and in neighborhoods with majority minority populations, allowing for cash memberships, and discounting membership fees for food stamp recipients [26]. Approximately one-third of the bikeshare stations have been situated in lower-income neighborhoods and the program estimates that approximately 8% of residents aged 18- < 65 living in those lower-income neighborhoods have used the program [27].

We examined whether physical activity increased over time, among Philadelphia residents who joined bikeshare. We hypothesized that after approximately 12-months of follow-up, within-person total moderate and vigorous activity would increase among participants who had higher use of the program. Further, we tested whether this association differed by participant socio-economic disadvantage and race/ethnicity. Because some bikeshare members may become motivated to use a personal bike after joining the program [7, 28], we also hypothesized that increased use of a bike (bikeshare or personal bike) would be associated with increases in total moderate and vigorous activity.

## Methods

Between September 2015 and December 2017, a convenience sample of new bikeshare members enrolled in the Drexel Neighborhood Transportation Study (DNTS). Enrollment and follow-up survey collection were conducted during months favorable for biking (mid-March to the beginning of December) and was facilitated by the bikeshare vendor, Bicycle Transit Systems (BTS).

### Recruitment and eligibility

After paying for a bikeshare membership online, new members received the DNTS URL. New members who clicked on the weblink, were screened for eligibility. Briefly, inclusion criteria were aged ≥ 18, current resident of Philadelphia, unlikely to move out of Philadelphia in the next two years, willing to participate in the multi-year questionnaire periods, and no or low use of bikeshare previously. Details on eligibility and enrollment are in Supplement Fig. 1/Text.

### Assessment of total physical activity levels

In order to assess total physical activity, we used a modified version of the International Physical Activity Questionnaire (IPAQ-L) [29, 30]. This instrument has been widely used [31] and found to have acceptable measurement properties, at least as good as other established self-reports [29, 31]. More information about data collection is published separately [32] and is in Supplement Text. Briefly, total physical activity was represented by past 7-day self-reported activity for work, leisure, and transportation.

#### Total activity minutes

Within ‘moderate’ and ‘vigorous’ classifications, *total 7-day activity minutes* were computed for type of activity as well as total 7-day activity. Total 7-day moderate or vigorous physical activity (MVPA) was computed by adding moderate activity minutes per week + (2*vigorous activity minutes per week) [33].

We used two measures from total MVPA minutes per week, one measure that included walking minutes (AKA MVPA with walking”) and another measure that excluded walking (AKA non-walking MVPA).

The rationale for excluding walking from ‘moderate’ activity are the following: 1. Virtually all participants reported frequent transportation walking (> 85%) and walking accounted for the largest share total physical activity reported. Inclusion of walking makes it very difficult to detect changes in activities that represent a much smaller share of moderate or vigorous intensity activity, namely biking (Supplement Table 1). 2. Substitution of walking with cycling could have physical activity benefits due to cycling generally having higher MET values. Walking is generally assumed to have MET values of 3.5 to 4.0, whereas cycling is assumed to have MET 6.0 to 8.0 [34].

The primary rationale for including walking within MVPA is that walking is commonly included in the summary measures of MVPA [29]. The secondary rationale is that informing issues of substitution is not possible without examining total activity including walking as walking may decrease when participants start biking.

#### Classifying *‘inactive’*

We created a binary variable to indicate if the participant was inactive/low-activity. The substantive rationale for deriving a variable for inactivity/low-activity was to assess whether bikeshare can activate lower-activity populations. We defined inactive based on total MVPA levels being in the lowest quartile of the sample distribution. The methodological rationale for classifying activity based on the observed ranked distribution in the sample is that self-reported behaviors have low precision and the absolute value of minutes of activity is not very reliable [35]. Regarding the IPAQ specifically, the instrument is known to over-report activity minutes, particularly moderate-intensity activity [36]. Yet, within-sample relative values can successfully reflect within sample differences (i.e., those with higher relative values truly have higher activity levels than others and vice versa [37]). Other work has reported that when within-sample ranked values are used, self-report activity minutes aligns with ranked values for direct measures (such as accelerometry [36, 37]).

Two binary measures were derived for the lowest quartile in past 7 day MVPA. For non-walking MVPA, the lowest quartile corresponded to not engaging in at least 10 min of MVPA (hereafter referred to as ‘inactive’ for non-walking MVPA, and all other values will be referred to as ‘more active’). For MVPA with walking, the lowest quartile corresponded to not engaging in at least 150 min of MVPA with walking (hereafter referred to as ‘inactive’ for MVPA with walking, and all other values will be referred to as ‘more active’).

### Outcome – change in activity

First, we examined within-person change in 7-day MVPA (non-walking MVPA minutes and MVPA with walking) as a continuous variable; see modeled treatment of this variable in Statistical analysis. Second, we created a categorical change variable describing change in activity status (using ‘active’ vs. ‘inactive’ described above according to whether MVPA was non-walking or with walking). Three categories were created: *stayed the same* (no change in activity status, in other words not active at baseline and follow-up, or active at baseline and follow-up), *became inactive* (active at baseline but inactive at follow-up), and *became more active* (inactive at baseline but active at follow-up).

### Exposure

#### Bikeshare program use

##### Overview

Bikeshare trip data came from the City’s vendor, BTS. During the survey, participants granted us permission to link their survey to their bikeshare trips (trip date, distance, and approximate minutes). The vendor linked the two data sources using name, email, or cell phone. All member trips were included from the start of bikeshare (April 2015) through the end of cohort follow-up (December 2018). Participants’ bikeshare data was used to determine program use (days and trips) before their baseline survey and after their baseline survey.

##### Exposure – change in bikeshare use

First, we measured bikeshare use as a *continuous variable* (bikeshare days during the past 365 days at follow-up minus baseline). This (untransformed) continuous exposure measure was used as one of the exposures in the main analyses.

Second, we created a *categorical change* variable with three categories: 1. no bikeshare use during the past year, 2. ‘low use’ during the past year, defined as used bikeshare 1- < 15 days, and 3. ‘higher use’ during the past year, defined as used bikeshare at least 15 days. A 15-day threshold was chosen because it was close to the median number of days that the cohort used the program at follow-up and this inflection point was suggested by diagnostics (see Supplement Fig. 1). Bikeshare program data were collected for the entire study period but within a year, the distribution of biking trips occurred most during the 4–5 months when the weather is most favorable for biking in Philadelphia (and these months also aligned with the months of survey data collection). Assuming 15 trips were equally distributed across 5 months, this would mean that bikeshare exposure would be 3 times per month. We anticipated somewhat infrequent program use based on infrequent bikeshare use documented by others [38, 39].

#### Any bike use – bikeshare or personal bike

Because bikeshare may motivate members to engage with personal biking, including purchasing a personal bike, or increasing baseline use of a personal bike [28], a third exposure was *new use of any type of bike* (bikeshare or personal bike). We derived this from a combination of program data and survey responses regarding personal bike use and focused on past 30 day (AKA ‘recent use’) use of any type of bike.

We classified recent use of any bike into three categories: 1. no past month biking at follow-up, 2. past month biking at baseline and follow-up, 3. past month biking only at follow-up (not baseline). Category 1 included two groups: i. no past month biking at baseline or follow-up, and, ii. past month biking at baseline but not follow-up (a small number of participants were in this group thus could not serve as a stand-alone exposure category).

#### Additional variables used to control for baseline bike use

To control for persons who had some prior exposure to bikeshare and/or city biking, we derived two separate baseline variables for multivariable adjustment. The first variable was past 30 day use of any bike ≥ 6 trips and the second variable was bikeshare use for ≥ 6 trips at any point prior to baseline survey. Bike ‘trips’ was used in order to reduce collinearity with the main exposure variables (derived from bike ‘days’) and six trips was used as an indicator threshold because it was the median among participants who used the program prior to baseline.

### Disadvantaged status

To control for socio-economic disadvantage (hereafter referred to as ‘disadvantage’) and assess whether program use affected physical activity differently by disadvantaged status, we derived an indicator for disadvantage using educational attainment, employment/ occupational status, income and number of persons supported by the reported income (see Supplement Text).

### Secondary data: neighborhood bikeshare stations, roadway bikeability, and weather

Participant residential addresses were geocoded and spatially linked to *bikeshare station* locations and roadway *bikeability* (see Supplement Text). Weather data came from the National Climactic Data Center [40]. We derived past-7-day maximum daily temperature and mean daily precipitation for each participant, using their survey date as their index date. Weather conditions are important to control for as unsuitable weather is known to deter biking [41] and in the Philadelphia area, weather is highly variable. Daytime temperatures average from mid-20 °F (-3.8 °C) in the winter to upper-90 °F (35 °C) in the summer [40].

### Statistical analysis

Descriptive statistics (mean, proportions) were used to describe the cohort. Continuous outcomes were modeled using negative binomial regression. Negative binomial regression is a generalization of the Poisson distribution and is suitable to model non-negative count data with overdispersion [42]. To improve interpretation, regression-adjusted coefficients were exponentiated and interpreted as percentages. The continuous outcome was total MVPA at follow-up, conditional on baseline MVPA (AKA the lagged dependent variable or ‘regressor variable method’ [43–45]). A three-category outcome was modeled via multinomial logistic regression. The models computed adjusted odds ratios for ‘became *inactive*’ relative to ‘stayed the same’ (no change in physical activity status) and adjusted odds ratios for ‘became *active*’ relative to ‘stayed the same’.

Main results are shown separately for non-walking MVPA and MVPA with walking (see justification in Assessment of total physical activity levels).

Model exposures were past year use of the bikeshare program operationalized in two ways: change in number of days used program (continuous measure of follow-up minus baseline), and categorical change in number of days (0 days, 1- < 15 days, 15 or more days). Additionally we used an exposure that incorporated bikeshare and personal bike: any recent bike use (past month).

Model control variables were determined a priori based on diagrams, and literature [46]. Sequential adjustment added control variables into the model in stages. Results did not dramatically change across the stages of adjustment and thus will be shown only for the fully adjusted model. We adjusted for person characteristics (age, gender, race/ethnicity, household composition [alone, with other adult, with children], disadvantage [baseline disadvantage and change in disadvantage over follow-up], car ownership, health status at follow-up [chronic disease status and health problem in past 30 days]), weather variables (past 7 day temperature and precipitation, both classified into quartiles), proxies of biking infrastructure around the residence (density of bike share stations, and percent of roadways that had low bikeability), and distance between residence and city hall as a rough proxy for whether each participant was geographically proximal to a high concentration of amenities/potential destinations. Additionally, we adjusted for baseline bike use.

In order to assess differences by disadvantaged status in the effect of bikeshare use on changes in activity, the product of higher program use and disadvantage status was entered into the fully adjusted model and the interaction Type III test statistic *p*-value was reported. We followed the same steps to assess whether there were differences by race/ethnicity White non-Hispanic vs. non-White (i.e., non-White Hispanic and non-White non-Hispanic) on the effect of bikeshare on changes in activity status.

### Sensitivity analysis

While this study lacked detailed data to fully assess substitution of one type of past 7-day MVPA for another, we nevertheless employed a few methods to roughly inform substitution. First, regarding substituting leisure activity with biking/bikeshare, we examined results after adjusting for past 7-day leisure. Second, regarding substituting walking activity with biking/bikeshare, we present main results for non-walking MVPA and contrast them with results for MVPA with walking. Additionally, in order to categorically remove potential substitution of walking with another MVPA type, we present results for non-walking MVPA after excluding participants whose 7-day walking for transport declined. (Supplement Table 2).

Additional sensitivity analyses evaluated results after removing participants who did not use bikeshare in the past year (Supplement Table 3 and Supplement Table 4).

## Results

### Cohort demographics

Baseline median age of the cohort was 30 (interquartile range [IQR] 26–36), race/ethnic proportions were 60% White non-Hispanic, 18% Black and 6% Hispanic. One-third were classified as being disadvantaged (Table 1) and 63% of the cohort had per capita incomes < $35,000. The cohort’s race/ethnicity profile was a good representation of residents in the bikeshare service area, however, the cohort had higher income/education (determined from Census [47]).

**Table 1:** Demographics, by changes in activity status at follow-up (stayed the same, became inactive, became more active). *N* = 1031

				**Activity status at follow-up **^**a**^					
		**Total**		**Stayed same**		**Became inactive**		**Became more active**	
		N	Column %	N	Row %	N	Row %	N	Row %
		1031	100%	756	73.3%	118	11.4%	157	15.2%
**Characteristic**				N	Column %	N	Column %	N	Column %
Age	18 to < 30	513	50%	369	49%	59	50%	85	54%
	30 to < 40	321	31%	250	33%	40	34%	31	20%
	40 and over	197	19%	137	18%	19	16%	41	26%
Gender	Male	415	40%	309	41%	42	36%	64	41%
	Female	616	60%	447	59%	76	64%	93	59%
Race/Ethnicity	White (non-Hispanic)	620	60%	470	62%	68	58%	82	52%
	Black (non-Hispanic)	184	18%	120	16%	29	25%	35	22%
	Latino/Hispanic	61	6%	46	6%	6	5%	9	6%
	Asian, Pacific Islander, or South Asian	150	15%	109	14%	11	9%	30	19%
	Other (Native Am., Middle Eastern, Mixed)	16	2%	11	1%	4	3%	1	1%
Disadvantaged socio-economic status ^b^	Yes	309	30%	233	31%	47	40%	61	39%
Income (per capita in household)	Total, mean (std)	$47,100	$33,300	$46,400	$33,800	$41,000	$30,700	$38,100	$31,100
	Among students, mean (std)	$29,100	$22,800	$28,000	$20,000	$24,100	$17,900	$24,500	$15,100
	Among non-students, mean (std)	$53,300	$34,100	$53,300	$35,300	$48,400	$32,300	$44,800	$34,700
Education	Years of schooling, mean (std)	16.2	1.9	16.2	2.0	15.9	2.0	16.0	2.1
	Has Bachelor's degree (4-year college)	809	78%	641	85%	96	81%	128	82%
Employment and student status ^c^	Percent reporting as student	296	29%	208	28%	36	31%	52	33%
	Percent reporting as full time workers	629	61%	480	63%	62	53%	87	55%
Household composition	Lives alone	296	29%	214	28%	38	32%	44	28%
	Lives with other adults	549	53%	416	55%	53	45%	80	51%
	Children in household	186	18%	126	17%	27	23%	33	21%
Health status variables ^d^	Chronic illness	246	24%	166	22%	39	33%	41	26%
	Past month, 7 + days physically/mentally unwell	281	27%	166	22%	39	33%	41	26%
Length of time lived in Philadelphia (years)	Median (25th and 75th percentile)	4	(1,14)	4	(1–12)	5.5	(1.5–21)	4	(1–21)
Remained in same ZIP Code, baseline to follow-up	Yes	828	80%	614	81%	90	76%	124	79%

*Abbreviations*: *MVPA* Moderate or vigorous physical activity, *Min* Minutes, *Std* Standard deviation

^a^ “Inactive” in this table is defined as less than 10 min per week of non-walking MVPA. "More active" refers to not inactive

^b^ Disadvantaged is defined in the Supplement

^c^ Employment and student status (not mutually exclusive)

^d^ Chronic illness refers to ever told by medical professional that had elevated blood pressure or cholesterol, or coronary artery disease, or diabetes

### Biking infrastructure, bike use, and mode of transport

Table 2 shows within-person changes in activity and factors that may support biking. The majority of the cohort lived within 400 m of a bikeshare station. Approximately 33% of the cohort owned a personal bike at baseline and this rose over follow-up to 40%. At baseline, 36% had used bikeshare at some prior point but had used it minimally (rode a median of 5 trips). At follow-up, *over the past 12 months*, 73% of the cohort used bikeshare. Among users, they took a median of 33 trips (IQR 9, 107), totaling approximately 20 min per day over a median 21 days (IQR 6, 67). At follow-up, 25% of the cohort *recently* used bikeshare (30 days prior to follow-up survey) and 42% recently used any type of bike (bikeshare or personal bike). At follow-up, 45% of participants reported that walking or biking was their main mode of transport. Among those who switched their main mode of transport to personal bike or bikeshare (*N* = 107), 47% switched from walking, 35% from public transit, and 18% from motor vehicle (details not shown in table).

**Table 2:** Unadjusted findings of within-person changes in bike infrastructure, bicycle and car ownership, main mode of transportation, bike use and total physical activity. *N* = 1031

		**N**	**Column %**
**Total**		1031	100%
**Bike infrastructure**			
Bikeshare station density nearby residence (within 400 m [0.25 mile])			
No stations within 400 m, both W1 and W2		210	20%
At least 1 station, both W1 or W2		691	67%
At least 1 station within 400 m in W2 only (not W1)		69	7%
At least 1 station within 400 m in W1 only (not W2)		61	6%
Low bikeability (within 800 m or [0.50 mile] of residence)			
Percent of roadway length that had high traffic stress, median (25th-75th percentile)		36%	(29%, 41%)
**Bike ownership and car ownership**			
Self-owned bike			
None, no bike at either W1 and W2		542	53%
Had personal bike, both W1 and W2		300	29%
Had personal bike, W1 only (not W2)		37	4%
New personal bike, W2 only (not W1)		152	15%
Automobile at residence (among those with license)			
None, no car at either W1 and W2		222	27%
Owns car, W1 and W2		562	68%
Owns car, W1 only (not w2)		46	6%
**Most common mode of transportation (self-report)**			
Motor vehicle			
Motor vehicle W1 and W2		186	18%
Motor vehicle W1, public transit in W2		40	4%
Motor Vehicle in W1, bike/walk in W2		56	5%
Public transit			
Public transit W1 and W2		191	19%
Public transit W1, motor vehicle in W2		48	5%
Public transit W1, bike or walk in W2		88	9%
Bike or walk			
Bike or walk W1 and W2		319	31%
Bike or walk in W1, motor vehicle in W2		47	5%
Bike or walk in W1, public transit in W2		56	5%
**Physical activity: Bicycle use**			
Recently used any bike (past 30 day personal or bikeshare ^a^)			
Didn't use any bike in W1 or W2		474	46%
Used any bike in W1 and W2		198	19%
Used any bike in W1 not in W2		124	12%
Used any bike in W2 not in W1		235	23%
Recently used personal bike (past 30 days)			
Didn't use a personal bike in W1 or W2		720	70%
Used personal bike in W1 and W2		102	10%
Used personal bike in W1 not in W2		88	9%
Used personal bike in W2 not in W1		121	12%
Used bikeshare, past month (30 days ^b^)			
Didn't use bikeshare in past month W1 or W2		673	65%
Use bikeshare in past month both W1 and W2		60	6%
Use bikeshare in past month in W1 not W2		101	10%
Use bikeshare in past month in W2 not W1		197	19%
Used bikeshare, past year (365 days ^b^)			
Didn't use bikeshare in past year W1 or W2		231	22%
Use bikeshare in past year both W1 and W2		291	28%
Use bikeshare in past year in W1 not W2		51	5%
Use bikeshare in past year in W2 not W1		458	44%
Change in past year bikeshare use ^b^			
No use, zero days		282	27%
Low use, 1—< 15 days		306	30%
Higher use, 15 + days		443	43%
**Physical activity: Past 7-day moderate and vigorous activity (MVPA)**			
Inactivity status *non-walking MVPA* (inactive defined as zero minutes MVPA in past 7 days)			
Inactive in both W1 and W2		110	11%
More active in both W1 and W2		646	63%
Inactive in W1, active in W2		157	15%
Inactive in W2, active in W1		118	11%
Inactivity status, *MVPA includes walking* (inactive defined as < 150 min in past 7 days)			
Inactive in both W1 and W2		83	8%
More active in both W1 and W2		731	71%
Inactive in W1, active in W2		102	10%
Inactive in W2, active in W1		115	11%
Biked for transport ≥ 10 min at a time (any type of bike, contributed to past 7 day moderate activity)			
None past 7 days (W1 and W2)		592	57%
Both W1 and W2		107	10%
W1 not W2		113	11%
W2 not W1		219	21%
Leisure activity ≥ 10 min at a time (past 7 days moderate or vigorous)			
None past 7 days (W1 and W2)		159	15%
Both W1 and W2		549	53%
W1 not W2		150	15%
W2 not W1		173	17%
Work activity ≥ 10 min at a time (past 7 days moderate or vigorous)			
None past 7 days (W1 and W2)		989	96%
Both W1 and W2		10	1.0%
W1 not W2		16	1.6%
W2 not W1		16	1.6%

*Abbreviations*: *W1* Refers to baseline (wave 1), *W2* Refers to follow-up (wave 2)

^a^ Derived from bikeshare program data and personal bike use reported in the survey

^b^ Derived from bikeshare program data

### Physical activity at baseline and follow-up

At follow-up, most participants reported walking (87%) and MVPA leisure-time physical activity (70%). Past 7 day MVPA transportation bicycling was reported by 32% and MVPA work activity by 2.5% (Table 2). The proportions engaging in those activities were very similar to baseline except that at baseline only 21% reported transportation bicycling.

Unadjusted change in physical activity is reported in Table 2 and in Supplement Text.

#### Adjusted effects of bikeshare program use on changes in physical activity

##### Change in non-walking MVPA

In adjusted analysis, increased days of program use was associated with more minutes of non-walking MVPA at follow-up (Table 3, outcome 1). For example, per 10 days of program use, continuous minutes of non-walking MVPA at follow-up increased by 3%, conditional on non-walking MVPA at baseline and adjusted for covariates (95% confidence interval [CI] 1% to 5%, P 0.01 [exposure A-i]). When past year program exposure was examined using categorical thresholds (exposure A-ii), lower use (1- < 15 days) was associated with fewer non-walking MVPA minutes at follow-up relative to no use (P 0.01) and higher use (≥ 15 days) was not associated with continuous non-walking MVPA at follow-up (P 0.8).

Adjusted results suggested a positive relationship between more days of program use and higher odds of transitioning from *inactive* to *more active* over follow-up (‘became more active’) and lower odds of transitioning from *active* to *inactive* over follow-up (‘became inactive’), relative to no change in activity status (Table 3 non-walking MVPA, outcome 2). For example, higher threshold users of the program (≥ 15 days vs. no use) had 80% higher odds of ‘became more active’ (odds ratio [OR] 1.80, 95% CI 1.05, 3.09, P 0.03 [exposure A-ii, outcome 2A]) and 36% lower odds of ‘became inactive’ (OR 0.64, 95% CI 0.35, 1.18, P 0.15 [exposure A-ii, outcome 2B]).

**Table 3:** Regression results for non-walking MVPA. Adjusted^a^ within-person differences in non-walking moderate or vigorous physical activity (MVPA) minutes and change in activity status (became more active, became inactive), according to number of days used the program and used any type of bike. *N* = 1031

	Negative binomial regression. Continuous outcome				Multinomial logistic regression. 3-category outcome (stayed the same 73.3%, became more active 15.2%, became inactive 11.5%)							
	Outcome 1. MVPA minutes at follow-up, controlling for baseline MVPA				Outcome 2A. Became more active vs. stayed the same^b^				Outcome 2B. Became inactive vs. stayed the same^b^			
		95% Confidence Interval				95% Confidence Interval				95% Confidence Interval		
	Exp(β)	Low	High	*P-value*	Exp(β)	Low	High	*P-value*	Exp(β)	Low	High	*P-value*
**A. Exposure to bikeshare**												
** i.** Exposure is continuous change in 10 days of program use ^c^												
	**1.29**	**1.26**	**1.31**	**0.014**	1.02	0.99	1.04	0.198	**0.94**	**0.89**	**0.99**	**0.029**
**ii.** Categorical exposure, past year change days used the program ^d^												
No use, zero days	Referent				Referent				Referent			
Low use, 1—< 15 days	**0.68**	**0.50**	**0.92**	**0.014**	1.45	0.83	2.52	0.189	1.54	0.88	2.71	0.133
Higher use, 15 + days	1.03	0.76	1.40	0.835	**1.80**	**1.05**	**3.09**	**0.033**	0.64	0.35	1.18	0.154
**B. Exposure to bikeshare or personal bike**												
**i.** Categorical exposure, change in recent bike use (past 30 day personal or bikeshare use) ^e^												
No bike use at follow-up	Referent				Referent				Referent			
Bike use at baseline + follow-up	**1.52**	**1.10**	**2.11**	**0.012**	0.93	0.51	1.69	0.819	**0.33**	**0.16**	**0.71**	**0.004**
New bike use at follow-up (not baseline)	**1.50**	**1.15**	**1.96**	**0.003**	1.04	0.67	1.61	0.848	**0.55**	**0.32**	**0.97**	**0.039**

^a^ Results adjusted for socio-demographics (age, sex, race/ethnicity, disadvantage, per capita income, household composition), number of cars, health status (presence of chronic illness, health status in past month), stayed at same residence, survey season, past 7 days weather, neighborhood biking infrastructure (stations, bikeability, distance to city hall), baseline bike use (personal or bikeshare)

^b^ “Inactive” in this table is defined as less than 10 min per week of non-walking MVPA. "More active" refers to not inactive

^c^ Past year program use at follow-up minus baseline. The model adjusted for all covariates listed above except for baseline program use; this aimed to improve interpretation, even though inference was unchanged

^d^ Bikeshare program use in the past 365 days between baseline and follow-up surveys had the following distribution: (1) no use *N* = 282 (27%), (2) one day to less than 15 days N = 306 (30%), (3) high use *N* = 443 (43%)

^e^ Any bike use in past 30 days had the following distribution: 1. no bike use at follow-up *N* = 598 (58%) (which was comprised of no bike use at baseline or follow-up [*N* = 474] + bike at baseline but not follow-up [*N* = 124]), 2. used bike at baseline and follow-up *N* = 198 (19%), and 3. used bike at follow-up but not at baseline *N* = 235 (23%)

##### Change in MVPA, walking included

In adjusted analysis, increased days of program use was not associated with more minutes at follow-up of MVPA with walking (Table 4, outcome 1, expβ 1.01, P 0.12) and was not associated with the odds of transitioning from *inactive* to *more active* over follow-up (Table 4, outcome 2, where categorical change was defined as at least 150 min in past 7-days of MVPA with walking). Similar to results in Table 4, when past year program exposure was examined using categorical thresholds (exposure A-ii), lower use (1- < 15 days) was associated with fewer non-walking MVPA minutes at follow-up relative to no use (P 0.01).

**Table 4:** Regression results with walking included in MVPA. Adjusted^a^ within-person differences in moderate or vigorous physical activity (MVPA, with walking) minutes and change in activity status (became active, became inactive), according to number of days used the program and used any type of bike. *N* = 1031

	Negative binomial regression. Continuous outcome				Multinomial logistic regression. 3-category outcome (stayed the same 79.0%, became active 9.9%, became inactive 11.2%)							
	Outcome 1. MVPA minutes at follow-up, controlling for baseline MVPA				Outcome 2A. Became active vs. stayed the same^b^				Outcome 2B. Became inactive vs. stayed the same^b^			
		95% Confidence Interval				95% Confidence Interval				95% Confidence Interval		
	Exp(β)	Low	High	*P-value*	Exp(β)	Low	High	*P-value*	Exp(β)	Low	High	*P-value*
**A. Exposure to bikeshare**												
**i.** Exposure is continuous change in 10 days of program use ^c^												
Continuous, per 10 days used program	1.01	1.00	1.02	0.116	0.95	0.90	0.96	0.064	1.00	0.96	1.05	0.858
**ii.** Categorical exposure, past year change days used the program ‡												
No use, zero days	Referent				Referent				Referent			
Low use, 1—< 15 days	**0.78**	**0.65**	**0.95**	**0.013**	1.16	0.64	2.13	0.621	1.18	0.65	2.14	0.587
Higher use, 15 + days	0.96	0.79	1.15	0.647	0.83	0.44	1.55	0.556	1.21	0.67	2.18	0.537
**B. Exposure to bikeshare or personal bike**												
**i.** Categorical exposure, change in recent bike use (past 30 day personal or bikeshare use)												
No bike use at follow-up	Referent				Referent				Referent			
Bike use at baseline + follow-up	1.20	0.98	1.48	0.073	0.56	0.27	1.19	0.134	0.79	0.38	1.63	0.515
New bike use at follow-up (not baseline)	**1.23**	**1.05**	**1.45**	**0.011**	0.63	0.36	1.12	0.113	1.11	0.67	1.84	0.688

^a^ Results adjusted for socio-demographics (age, sex, race/ethnicity, disadvantage, per capita income, household composition), number of cars, health status (presence of chronic illness, health status in past month), stayed at same residence, survey season, past 7 days weather, neighborhood biking infrastructure (stations, bikeability, distance to city hall), baseline bike use (personal or bikeshare)

^b^ “Inactive” in this table is defined as less than 150 min per week of MVPA including walking. "More active" refers to not inactive

^c^ Past year program use at follow-up minus baseline. The model adjusted for all covariates listed above except for baseline program use; this aimed to improve interpretation, even though inference was unchanged

#### Adjusted effects for recent bike use and changes in physical activity

##### Change in non-walking MVPA

Adjusted results suggested a positive relationship between recent bike use (past 30 days of any type of bike, bikeshare or personal) and an increase in minutes of non-walking MVPA. For example, relative to no recent bike use at follow-up, new bike use at follow-up was associated with 50% increased minutes of non-walking MVPA at follow-up (expβ 1.50, 95% CI 1.15 to 1.96, P 0.003 [Table 3, outcome A, exposure B-i]). The magnitude of effect was similar for participants who used any type of bike at baseline and continued to use a bike at follow-up. However, new bike use was not associated with ‘became more active’ (vs. stayed the same, *P* > 0.8 [exposure B-i, outcome 2A]) even though it appeared to be protective of converting from active to inactive. For example, any type of bike use was associated with at least 45% lower odds of ‘became inactive’, relative to no change in status (new bike use at follow-up but not at baseline P 0.039, continued use of a bike at follow-up P 0.004 [exposure B-i, outcome 2B]).

##### Change in MVPA, walking included

Adjusted results suggested a positive relationship between recent bike use (past 30 days of any type of bike, bikeshare or personal) and an increase in minutes of MVPA with walking. For example, relative to no recent bike use at follow-up, new bike use at follow-up was associated with 23% increased minutes of MVPA with walking at follow-up (expβ 1.23, 95% CI 1.05 to 1.45, P 0.01 [Table 4, outcome A, exposure B-i]). The magnitude of effect was similar for participants who used any type of bike at baseline and continued to use a bike at follow-up. However, new bike use was not associated with ‘became more active’ or ‘became inactive’, relative to no change in status (Table 4 exposure B-i, outcome 2A and 2B, where categorical change was defined as at least 150 min in past 7-days of MVPA with walking).

### Heterogeneity

We found no evidence that the effect of higher bikeshare program use on physical activity was different for participants who were disadvantaged (compared to not disadvantaged) or for participants who were non-White non-Hispanic or Hispanic (compared to participants who were White non-Hispanic). Product term interactions entered into regression were all *P* > 0.3 (data not shown).

### Sensitivity analyses

A set of sensitivity analyses focused on whether the positive effect of program use on non-walking MVPA could be explained as simply the effect of substituting one type of MVPA at baseline with biking/bikeshare MVPA at follow-up. Sensitivity analyses confirmed that changes in MVPA could be attributed to increases in past 7 day transport biking, not leisure activity. After adjusting for change in past 7 day leisure, inference was unchanged (the effect of program days on total 7-day non-walking MVPA remained *P* < 0.007) whereas, after adjusting for change in past 7-day transport biking, inference changed to null (the effect of program days on total 7-day MVPA was not evident, *P* < 0.35). Sensitivity analyses also suggested that changes in continuous non-walking MVPA could be attributed to increases in past 7-day transport biking, rather than declines in walking; seen in the similarity of results when comparing Table 3 to Supplement Table 2, after excluding participants whose 7-day walking for transport declined.

We also assessed whether results changed after removing participants who did not use bikeshare in the past 12 months (Supplement Table 3 and Supplement Table 4); results were similar to or stronger than results shown in main Tables 3 and 4.

## Discussion

This evaluation enrolled a cohort of new bikeshare members and reported on changes in total physical activity levels after one year. As expected, bikeshare use increased dramatically after participants’ baseline survey and personal bike ownership also increased. Overall, more days using the bikeshare program was associated with a slight within-person increase in non-walking MVPA minutes at follow-up (consistent across a variety of sensitivity analyses). Bikeshare’s ability to convert people from inactive to more active ('became more active') was only evident when cohort members reached a threshold of higher use of bikeshare (at least 15 days, relative to no bikeshare use) and when MVPA excluded walking. Favorable impacts on change in MVPA were observed when bikeshare exposure was broadened to include recent use of any type of outdoor bicycle (personal bike or bikeshare). Finally, results were consistent for socio-demographic subgroups, advantaged vs. disadvantaged participants and for White vs. non-White participants.

No prior studies have examined within-person change in total physical activity in response to bikeshare. Research on within-person changes in MVPA in response to other types of bike/pedestrian infrastructure has reported null findings [48–50], in part because the studies were not powered to detect small effects. An exception was Goodman’s et al. [51] study of approximately 1500 UK adults that reported within-in person post–pre 10.5 weekly minute change in MVPA among residents who lived near newly constructed street crossings and bridges intended to facilitate walking/cycling behaviors (relative to residents who did not live near the new infrastructure).

Our adjusted results found that new bikeshare members experienced a small increase in their weekly MVPA levels (net of their baseline level). For example, within the cohort, per 10 days of program use during the past year, non-walking MVPA at follow-up increased by approximately 6 additional weekly minutes (extrapolated from a 3% to 4% change from baseline median weekly non-walking MVPA in the cohort, observed in main results and sensitivity analyses [Table 3, Supplement Table 2, Supplement Table]), a change that is unlikely to confer any health benefits. However, for higher program users, there could be larger gains in weekly non-walking MVPA; for example if we extrapolate to 100 days of program use, it could yield approximately 60 additional minutes in weekly MVPA. Additionally, our results suggest bikeshare spillover effects on new use of any type of bike which could confer health benefits. Uptake of recent bike use at follow-up (personal or bikeshare) was consistently associated with at least 50% net increase in within-person MVPA (non-walking and with walking) which could yield approximately 110 additional minutes in weekly MVPA.

Importantly, we found no evidence that bikeshare effects on within-person change in physical activity differed by participant socio-economic disadvantage or by participant race/ethnicity (non-White non-Hispanic vs. others). Based on our work, the bikeshare program in Philadelphia, specifically designed with an equity focus, did not exacerbate socio-economic and race/ethnic inequalities in physical activity.

Adults who are still relatively healthy, but are inactive, are a key target population for public health interventions [52]. We anticipated that our study would observe increases in total activity among cohort members who are insufficiently active at baseline. However, results from the current evaluation were inconsistent regarding bikeshare’s potential for this. One operationalization of the data suggested that using the program at least 15 days in the past year activated those who were inactive at baseline (80% higher odds of becoming more active, relative to no change in activity status and when defining inactive based on non-walking MVPA). However, most other operationalizations found no clear effect of bikeshare on activating those who were inactive at baseline.

For bikeshare to have a substantively significant effect on physical activity levels, cities will need to address issues associated with low program use. In our cohort, only 25% of members used the program in the 30 days prior to their one-year follow-up survey. Over the year follow-up, some members switched to personal bike (or combined personal and bikeshare use); however, close to 60% of the cohort did not recently bike outdoors. Bikeshare programs in other cities have also reported low frequency of bikeshare use, approximately 4 trips per month (median across members) [38, 39, 53], which roughly aligns with median use found in our study. One of the reasons for low frequency of program use (which applies to street biking in general) is inadequate infrastructure for safe biking [54–56]. While Philadelphia has 28 miles of bicycle lanes [57], few of the lanes are protected from vehicles. Specific to our cohort, nearly 40% of the roadways around participants’ residences had higher traffic stress. Station proximity is another important motivator for bikeshare frequency [6, 10]. While the Philadelphia bikeshare program service area covers an impressive 20 square kilometers (8 square miles) [24], 20% of the members in our cohort did not reside close (400 m) to a bikeshare station.

Our study lacked detailed time/ compositional data [13] on all types of sedentary behavior and physical activity, thus, we cannot determine with certainty whether or not new biking was substituting for some other type of MVPA. Nevertheless, results suggested that the effect of joining bikeshare on net change in non-walking MVPA was due to increases in transport cycling using a combination of bikeshare and personal bikes (see findings from sensitivity analysis and descriptive findings that past 7 days transport cycling increased approximately 10 percentage points, whereas other types of past 7 day non-walking MVPA remained quite stable). On the other hand, our results also suggest some substitution of baseline walking with biking at follow-up. On average, walking minutes decreased slightly by the follow-up period and when the outcome was change in MVPA *with walking*, the overall effect of bikeshare was only apparent when the exposure was new use at follow-up of any type of bike (bikeshare or personal bike), but not when the exposure was limited to days used bikeshare. While substitution is an important issue, substitution of lower MET activity (walking) with a higher MET activity (cycling) could still have physical activity benefits [34].

### Strengths

Several novel aspects of the methodological components strengthen this evaluation. First, this is the first large evaluation to date that prospectively assessed within-person changes in MVPA among bikeshare members. Second, by linking the cohort survey data to their bikeshare trip data we utilized objective information on bikeshare use; and we also included multiple measures of bicycle use (personal and bikeshare). Third, cohort participants were selected based on their willingness to join bikeshare (i.e., they were all willing to bicycle on the street). Thus, our assessment of changes in total physical activity levels in response to lower or higher use of bikeshare would not be severely confounded by attitudinal and behavioral factors related to willingness to bike on the street [19]. Fifth, our evaluation was able to examine whether the effects of bikeshare on physical activity differed by disadvantaged status and race/ethnicity of the member.

### Limitations

The following limitations are worth noting. *First*, the study design lacked a clear control group. We examined the impact of joining a bikeshare program on physical activity, using a convenience sample where dose of program use was the exposure and within-person change in MVPA was the outcome. Future studies could consider neighborhood-based sampling where participants are recruited before bikeshare stations are placed in a neighborhood, participants are followed over time to observe who joined and did not join bikeshare and then within-person changes in physical activity are assessed. *Second*, results are likely generalizable to urban populations who are willing and physically capable of getting on a bicycle, but may not be generalizable to others. *Third*, this evaluation relied on self-reports of physical activity. Self-reports are critical for gaining insight into total physical activity levels of populations, but have well-known limitations, specifically, recall bias and inaccuracies assessing the absolute level of physical activity. Nevertheless, the survey instrument we used has been widely used for physical activity measurement [31], including assessing changes in bicycle use [10, 58, 59], and has acceptable measurement properties, at least as good as other survey instruments [29, 31]. Available data on leisure physical activity levels in Philadelphia roughly align with the cohort’s self-reported physical activity levels [60]. *Fourth*, for many in the cohort, there was a temporal mis-match between exposure to the program over a number of months and past 7-day measurement of physical activity. If the program effects are true, the program likely had a lagged effect. *Fifth*, our survey did not collect sufficiently detailed information on personal bike use throughout the past year to understand the full contribution of personal bike use to net change in MVPA.

## Conclusions

Our evaluation points to bikeshare as a facilitator of cycling in general and modest increases in non-walking MVPA. Program use was associated with small increases in non-walking MVPA and, at higher levels of program use, results suggested that bikeshare motivated some participants to transition from inactive at baseline to being more active at follow-up. Nevertheless, for the city to generate higher uptake and frequency of bikeshare (which could lead to higher MVPA and activate residents who are generally inactive), it will be important to increase the safety of bike riding, as well as, enhance the convenience and accessibility of bikeshare. Philadelphia plans to maintain low-income subsidies as well as extend the service area and double their bikeshare fleet size by 2026 (including making one-half electric-assisted bicycles) [61, 62]. These enhancements could result in increased ridership and frequency of use which could have positive effects on activating city residents.

## Supplementary Information

**Additional file 1: Supplement Figure 1.** Sample recruitment, enrollment, and retention. **Supplement Methods Text.** Details on measuring total physical activity levels and other variables. **Supplement Table 1.** Distribution of continuous measures of past 7-day activity. **Supplement Table 2.** Regression results for non-walking MVPA, subset to participants who used bikeshare in past year. **Supplement Table 3.** Regression results for non-walking MVPA, excluded participants who decreased their walking minutes at follow-up. **Supplement Table 4.** Regression results with walking included in MVPA, subset to participants who used bikeshare within the past year. **Supplement Figure 2.** Within-person change in total physical activity minutes as a function of within-person change in bikeshare use.

## Data Availability

A copy of the online survey instrument will be provided upon request to the corresponding author (programmed for Qualtrics Survey Software). Participant data are not publicly available due to human subjects research protections but qualified academic researchers may send data requests to the corresponding author for review. All use of the data would be subject to confidentiality and data-use agreements.
