# Supplementary material for: Changes in physical activity after joining a bikeshare program: a cohort of new bikeshare users
Source: Int J Behav Nutr Phys Act. 2022 Oct 4;19:132. doi: 10.1186/s12966-022-01353-6 (PMC9533574; doi:10.1186/s12966-022-01353-6)
Supplement: Supplementary file 1 — Additional file 1: Supplement Figure 1. Sample recruitment, enrollment, and retention. Supplement Methods Text. Details on measuring total physical activity levels and other variables. Supplement Table 1. Distribution of continuous measures of past 7-day activity. Supplement Table 2. Regression results for non-walking MVPA, subset to participants who used bikeshare in past year. Supplement Table 3. Regression results for non-walking MVPA, excluded participants who decreased their walking minutes at follow-up. Supplement Table 4. Regression results with walking included in MVPA, subset to participants who used bikeshare within the past year. Supplement Figure 2. Within-person change in total physical activity minutes as a function of within-person change in bikeshare use. [file 12966_2022_1353_MOESM1_ESM.pdf]

Supplement Files

TITLE: Changes in physical activity after joining a bikeshare program: a cohort of new bikeshare users

AUTHORS:

Amy H. Auchincloss, Yvonne L. Michael, Saima Niamatullah, Siyu Li, Steven J. Melly, Meagan L. Pharis, Daniel Fuller

CORRESPONDING AUTHOR:

Amy H. Auchincloss, Dornsife School of Public Health, Drexel University, Philadelphia, Pennsylvania USA
Email: aha27@drexel.edu

Contents

Supplement Figure 1. Sample recruitment, enrollment and retention

Supplement Methods Text. Details on measuring total physical activity levels and other variables.

- Assessment of total physical activity levels
- Disadvantaged status
- Secondary data: Neighborhood bikeshare stations and roadway bikeability
- Supplemental text for unadjusted change in physical activity

Supplement Table 1. Distribution of continuous measures of past 7-day activity.

Supplement Table 2. Regression results for non-walking MVPA, subset to participants who used bikeshare in past year.

Supplement Table 3. Regression results for non-walking MVPA, excluded participants who decreased their walking minutes at follow-up.

Supplement Table 4. Regression results with walking included in MVPA, subset to participants who used bikeshare within the past year.

Supplement Figure 2. Within-person change in total physical activity minutes as a function of within-person change in bikeshare use.

References

SUPPLEMENT FIGURE 1. SAMPLE RECRUITMENT, ENROLLMENT AND RETENTION

Sample size calculation. At baseline, the study planned to enroll 1370 new bikeshare members in order to test our main hypothesis that the program would have a net increase in MVPA. By the end of the study, we expected to retain approximately 700 participants (based on other work, we anticipated only 50% retention). The target sample size assumed beta 80% and alpha 0.05, and being able to detect a fairly small effect size (≥ 0.15 standard deviation) assuming moderate to high correlation between measures (rho 0.5) and 2 repeat measures. Note that effect sizes ≤ 0.2 are considered small effects. In actuality, we enrolled 1206 study participants (slightly under-target) and by the end of the study we retained 1031 participants (over-target), which represented an 85% retention rate (see **Figure S1**).

Recruitment. After paying for a bikeshare membership online, new members were directed to a 'payment received thank you' webpage and were sent email confirmation of their new membership. The DNTS URL was shown on the 'thank you' webpage and at the bottom of the email. By clicking on the weblink, the user was taken to an external website maintained by the study. The study webpage included a description of the survey and FAQ. The survey took approximately 20 minutes to complete and participants were compensated for their time ($20 for baseline, $25 for the one-year follow-up, and $30 for the two-year follow-up). See flow diagram **Figure S1**.

Eligibility criteria. Enrollment eligibility criteria were designed to facilitate follow-up and ensure low bikeshare exposure at baseline: 1. aged ≥18, 2. current resident of Philadelphia, 3. unlikely to move out of Philadelphia in the next two years, 4. willing to provide contact information (email, phone, address) and participate in the questionnaire periods, and 5. not used bikeshare previously or had low use of bikeshare. Low use of bikeshare was defined in two ways: 1. Ever used bikeshare <5 times and not used in previous 7 days, or 2. ever used >=5 times but had not used in past month. Ineligible participants were asked to disclose their race/ethnicity and respond to a single physical activity question. We implemented multiple methods to authenticate online survey data and guard against repeat-responders [1]. Potential duplicates were flagged, further investigation was performed and if needed, authentication via phone was required to enroll. Each completed survey was manually reviewed as part of the quality control and assurance protocol.

Enrollment. We did not have direct access to the count of new members who joined the bikeshare each day. However, we used Google Analytics [2] to count the number of people who went to the survey webpage. Approximate 38% of people who went to the survey webpage, moved forward and completed the 'eligibility' screener (N= 3366 persons); among those, 36% (N=1206) were eligible and completed the main survey. Eligibles and ineligibles had roughly similar demographics and baseline value on a single physical activity question that was embedded in the enrollment screening tool (data not shown). The cohort's race/ethnicity profile was a good representation of residents in the bikeshare service area, however, the cohort had higher income/education (determined from Census [3]).

Retention over the follow-up period. Participants were invited to participate in the follow-up survey approximately 365 days after their baseline survey (mean days between baseline and completed follow-up survey = 368 [STD=10 days]). For the present analyses, from the 1206 participants who completed the baseline survey, we excluded an additional 106 participants (N=5 who did not agree to release their bikeshare trip data and N=101 who moved outside of Philadelphia during follow-up).

Validation checks and missing data. The evaluation's online questionnaire collected detailed socio-demographic data, bikeshare membership status, personal bike ownership and use, main mode of transport, car ownership, and physical activity and health-related factors, among other questions. The online survey had multiple validation checks (including summarizing their physical activity days/minutes and asking them if it 'sounded about right' or whether they wanted to revise their response). For the present analysis, there were no missing data. Demographic responses missing from the baseline survey were collected at the 12-month follow-up survey and/or study staff follow-up with participants via phone and email if answers to key questions were missing.

Supplement Figure 1. Flow diagram of recruitment and retention.

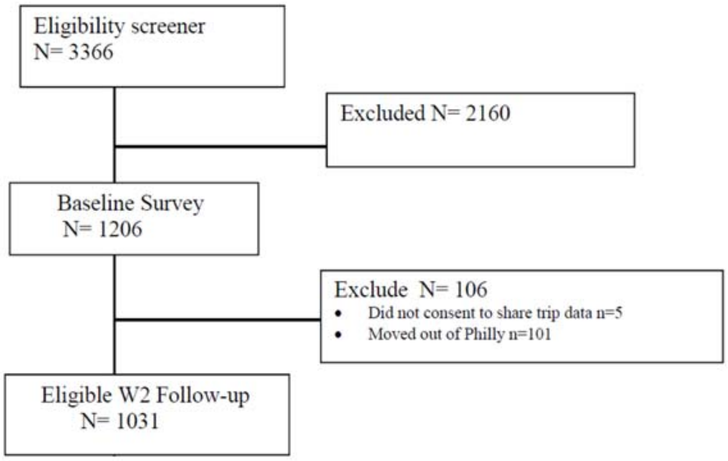

SUPPLEMENT METHODS TEXT

A. Assessment of total physical activity levels

Total physical activity levels were assessed using a modified version of the International Physical Activity Questionnaire (IPAQ-L) [4, 5]. This instrument has been widely used [6] and found to have acceptable measurement properties, at least as good as other established self-reports [4, 6]. More information about data collection is published separately.[7]

Modifications were made following recommendations by Rzewnicki et al. (2003) [8], for low-numeracy populations, and to accommodate online administration/response.

Total physical activity was represented by past 7-day self-reported activity along 3 domains, *work*, *leisure*, and *transportation*. What follows are details on collection of each of these domains. For *work* and *leisure* activity, participants were asked 'how many days' in past 7 days, 'how many minutes (or hours) on a typical day', and to only report activity that 'made you breathe harder than normal for at least 10 minutes at a time'. Additionally, for *work* activity, participants were asked to write-in the type of activity and job title. For *leisure* activity, participants used a pull-down menu to select from 63 activities (plus a write-in 'Other' option if not on the list). Job title and leisure activity selections were subsequently used to ascertain metabolic equivalent intensity level [9 , 10] in order to ascertain whether the activity required moderate or vigorous effort. For *transportation* activity, data were collected separately for transport bicycling and walking. Participants were asked days, trips, minutes each trip ('how many days' in past 7 days, on a typical day how many one-way trips and how many minutes each trip).

Additionally, for work activity, participants were asked to write-in the type of activity and job title. For leisure activity, participants used a pull-down menu to select from 63 activities (plus a write-in 'Other' option if not on the list). Job title and leisure activity selections were subsequently used to ascertain metabolic equivalent intensity level [9]. For transportation activity, data were collected separately for transport bicycling and walking. Participants were asked days, trips, minutes each trip ('how many days' in past 7 days, on a typical day how many one-way trips and how many minutes each trip).

B. Disadvantaged status

In order to control for socio-economic disadvantage (hereafter referred to as 'disadvantage') and also assess whether program use affected physical activity differently by disadvantaged status, we derived an indicator for disadvantage using educational attainment, employment/ occupational status, income and number of persons supported by the reported income. Details on this indicator are in **Supplement Text A**. Persons were classified as disadvantaged if they

had any of the following: 1. lower education (defined as adults aged >= 30 with less than 4-year college), 2. under-employed (participant selected "unemployed and seeking work" regardless of other employment/occupation categories selected), 3. lower income (reported <$35000 income per capita, approximately 200% of the federal poverty level). We took a conservative approach by excluding students aged <30 from criteria numbers 2 and 3. The rationale was that many students acquire little income but have access to family resources (allowances, housing costs covered, other living expenses paid by family). Our decision was supported by local research that found less than 30% of students from the three largest colleges in the bikeshare area come from families with lower-incomes [11].

Secondary data: Neighborhood bikeshare stations, roadway bikeability

We linked participant survey responses to bikeshare use data (described above) as well as other spatial data. Participant residential addresses were geocoded and spatially linked to *bikeshare station locations* and *roadway bikeability*.

Bike share station locations came from the bikeshare program. Circular buffers were calculated around each participant's residence. Density of bikeshare stations per land area around each participant's residence were derived for a 400m area; this distance represents a convenient walking distance to bikeshare [12] and other transit infrastructure [13, 14].

A *roadway bikeability index* was derived using an approach developed by others [15] and adapted to the Philadelphia context, using data from the city's offices of transportation and planning. We used the index to determine whether street segments had a high level of traffic-related road stress (AKA 'low bikeability') and then calculated the fraction of road segments around each participant's residence that had low bikeability. Low bikeability was for a 800m buffer area in order to correspond with perceptions of neighborhood bikeability.[16, 17]

C. Supplemental text for unadjusted change in physical activity

<u>Average change in physical activity across the cohort</u>

Across the cohort, there was a slight increase in minutes of MVPA without walking. At baseline, median minutes was 180 minutes, IQR 0-450 and 26% had no weekly minutes. At follow-up, median minutes of MVPA without walking was 225 (IQR 40-450) and 22% had no weekly minutes of MVPA.

<u>Within-person change in physical activity</u>

However, when considering within-person change in MVPA, most participants (73%) did not change their activity status (Table 2) and unadjusted median change in minutes was 0 (IQR -120, 180, not shown in tables). Older and disadvantaged members were more likely to become active; we found no other major differences across demographic groups (Table 1).

SUPPLEMENT TABLE 1. DISTRIBUTION OF CONTINUOUS MEASURES OF PAST 7-DAY ACTIVITY: MODERATE OR VIGOROUS PHYSICAL ACTIVITY (MVPA) MINUTES, WALKING, AND BIKING, IN THE FULL SAMPLE (N=1031) AND IN THE SUBSET OF PARTICIPANTS WHO USED BIKESHARE IN PAST YEAR (N=749)

	Entire sample<br>Past 7 day minutes of activity		Among those who used bikeshare >=1x in past year<br>Past 7 day minutes of activity	
	Percentile (P)		Percentile (P)	
	P50 (median)	P10, P25, P75, P90	P50 (median)	P10, P25, P75, P90
**MVPA without walking**				
Baseline	180	0, 0, 450, 750	180	0, 0, 440, 750
Follow-up	225	0, 40, 450, 768	220	0, 46, 440, 765
Change (follow-up minus baseline)	0	-350, -120, 180, 375	0	-350, -120, 175, 360
**MVPA with walking**				
Baseline	430	80, 210, 740, 1080	435	86, 220, 740, 1080
Follow-up	420	50, 180, 720, 1040	420	70, 200, 720, 1040
Change (follow-up minus baseline)	-15	-515, -220, 185, 440	-20	-520, -230, 180, 420
**Walk for transport**				
Baseline	160	0, 60, 350, 525	180	0, 80, 350, 525
Follow-up	130	0, 30, 280, 420	140	0, 40, 280, 420
Change (follow-up minus baseline)	-10	-330, -140, 60, 210	-20	-330, -140, 60, 200
**Bike for transport (any bike, either personal or bikeshare)**				
Baseline	0	0, 0, 0, 78	0	0, 0, 0, 60
Follow-up	0	0, 0, 40, 160	0	0, 0, 50, 160
Change (follow-up minus baseline)	0	-40, 0, 20, 120	0	-30, 0, 30, 120

SUPPLEMENT TABLE 2. REGRESSION RESULTS FOR NON-WALKING MVPA. EXCLUDED PARTICIPANTS WHO DECREASED THEIR WALKING MINUTES AT FOLLOW-UP. N=530.

Below shows results where participants were *excluded* if they decreased their walking minutes at follow-up. The intention of creating this subset was to remove those who could have substituted bikeshare for walking.

Results shown for adjusted within-person differences in **non-walking moderate or vigorous physical activity (MVPA) minutes** and change in activity status (became active, became inactive), according to number of days used the program and use any type of bike. N=503.

	Negative binomial regression<br>Continuous outcome				Multinomial logistic regression<br>3-category outcome (no change **70.6%**, became active **16.7%**, became inactive **12.7%**)							
	Outcome 1.<br>*Non-walking MVPA minutes at follow-up*<br>controlling for baseline MVPA				Outcome 2A.<br>*Became active*<br>vs. stayed the same				Outcome 2B.<br>*Became inactive*<br>vs. stayed the same			
		95% Confidence Interval				95% Confidence Interval				95% Confidence Interval		
	Exp(β)	Low	High	P-value	Exp(β)	Low	High	P-value	Exp(β)	Low	High	P-value
Models adjusted for socio-demographics, health staus, weather, neighborhood biking infrastructure, residence proximity to the center of the city, and baseline bike use *												
**A. Exposure to bikeshare**												
**i. Exposure is continuous change in program use #**												
Continuous, per 10 days used program	**1.04**	**1.00**	**1.07**	**0.046**	1.03	0.98	1.07	0.194	0.96	0.88	1.03	0.259
**ii. Categorical exposure, past year change in program use**												
**Past year, days used the program**												
No use, zero days	Referent				Referent				Referent			
Low use, 1 - <15 days	**0.64**	**0.38**	**1.06**	**0.084**	**2.17**	**1.01**	**4.69**	**0.05**	1.21	0.54	2.68	0.64
Higher use, 15+ days	0.97	0.59	1.60	0.905	**2.74**	**1.22**	**6.16**	**0.02**	0.71	0.30	1.71	0.45
**B. Exposure to bikeshare or personal bike**												
**i. Categorical exposure, change in recent bike use**												
**Recently used bike (past 30 day personal or bikeshare use)**												
No bike use at follow-up	Referent				Referent				Referent			
Bike use at baseline + follow-up	1.53	0.88	2.66	0.133	0.83	0.33	2.09	0.690	0.41	0.13	1.31	0.133
New bike use at follow-up (not baseline)	**1.62**	**1.04**	**2.52**	**0.033**	0.84	0.43	1.65	0.611	0.59	0.26	1.33	0.202

SUPPLEMENT TABLE 3. REGRESSION RESULTS FOR NON-WALKING MVPA. SAMPLE SUBSET TO PARTICIPANTS WHO USED BIKESHARE WITHIN THE PAST YEAR, N=749.

Below shows results after *excluding* those with zero days of bikeshare during the past 12 months, results were complimentary to results for the full sample but, in general, the magnitude of the effect was stronger.
Adjusted within-person differences in **non-walking moderate or vigorous physical activity (MVPA)** minutes and change in activity status (became active, became inactive), according to number of days used the program and use any type of bike.

	Negative binomial regression; Continuous outcome; Outcome 1. *MVPA minutes at follow-up* controlling for baseline MVPA				Multinomial logistic regression; 3-category outcome (no change **72.6%**, became active **16.4%**, became inactive **10.9%**); Outcome 2A. *Became active* vs. stayed the same				Outcome 2B. *Became inactive* vs. stayed the same			
		95% Confidence Interval				95% Confidence Interval				95% Confidence Interval		
	Exp(β)	Low	High	P-value	Exp(β)	Low	High	P-value	Exp(β)	Low	High	P-value
Models adjusted for socio-demographics, health staus, weather, neighborhood biking infrastructure, residence proximity to the center of the city, and baseline bike use *												
**A. Exposure to bikeshare**												
**i. Exposure is continuous change in program use** #												
Continuous, per 10 days used program	**1.036**	**1.015**	**1.058**	**0.001**	1.010	0.976	1.032	0.550	**0.935**	**0.884**	**0.989**	**0.020**
**ii. Categorical exposure, past year change in program use**												
**Past year, days used the program †**												
Binary variable, higher use (15+ days) vs. lower use (1-<15 days)	**1.518**	**1.185**	**1.943**	**0.001**	1.194	0.770	1.529	0.428	**0.385**	**0.230**	**0.643**	**0.000**
**B. Exposure to bikeshare or personal bike**												
**i. Categorical exposure, change in recent bike use**												
**Recently used bike (past 30 day personal or bikeshare use) ‡**												
No bike use at follow-up	Referent				Referent				Referent			
Bike use at baseline + follow-up	**1.709**	**1.205**	**2.424**	**0.003**	1.014	0.527	1.438	0.967	**0.223**	**0.092**	**0.541**	**0.001**
New bike use at follow-up (not baseline)	**1.593**	**1.200**	**2.116**	**0.001**	1.095	0.679	1.455	0.709	0.576	0.311	1.066	0.079

Supplement Table 3 Footnotes:
* Adjustment for age, sex, race/ethnicity, disadvantage, per capita income, household composition, number of cars, health status (presence of chronic illness, health status in past month), stayed at same residence, survey season, past 7 days weather, neighborhood biking infrastructure (stations, bikeability, distance to city hall), baseline bike use (personal or bikeshare)
# Past year program use at follow-up minus baseline. The model adjusted for all covariates listed above except for baseline program use; this aimed to improve interpretation, even though inference was unchanged.
† Bikeshare program use in the past 365 days between baseline and follow-up surveys had the following distribution: (1) no use N=282 (27%), (2) one day to less than 15 days N=306 (30%), (3) high use N=443 (43%). ‡ Any bike use in past 30 days had the following distribution: 1. no bike use at follow-up N=598 (58%) (which was comprised of no bike use at baseline or follow-up [N=474] plus bike at baseline but not follow-up [N=124]), 2. used bike at baseline and follow-up N=198 (19%), and 3. used bike at follow-up but not at baseline N=235 (23%).

SUPPLEMENT TABLE 4. REGRESSION RESULTS WITH WALKING INCLUDED IN MVPA. SAMPLE SUBSET TO PARTICIPANTS WHO USED BIKESHARE WITHIN THE PAST YEAR, N=749

Below shows adjusted within-person differences in **moderate or vigorous physical activity (MVPA, including walking)** minutes and change in activity status (became active, became inactive), according to number of days used the program and use any type of bike. After *excluding* those with zero days of bikeshare during the past 12 months, results were complimentary to results for the full sample but, in general, the magnitude of the effect was stronger.

	Negative binomial regression				Multinomial logistic regression							
	Continuous outcome				3-category outcome (no change **80.2%**, became active **9.5%**, became inactive **10.3%**)							
	Outcome 1. *MVPA minutes at follow-up* controlling for baseline MVPA				Outcome 2A. *Became active* vs. stayed the same				Outcome 2B. *Became inactive* vs. stayed the same			
		95% Confidence Interval				95% Confidence Interval				95% Confidence Interval		
	Exp(β)	Low	High	P-value	Exp(β)	Low	High	P-value	Exp(β)	Low	High	P-value
Models adjusted for socio-demographics, health staus, weather, neighborhood biking infrastructure, residence proximity to the center of the city, and baseline bike use *												
**A. Exposure to bikeshare**												
**i. Exposure is continuous change in program use** #												
Continuous, per 10 days used program	**1.017**	**1.005**	**1.030**	**0.007**	0.960	0.909	0.972	0.149	1.003	0.957	1.050	0.906
**ii. Categorical exposure, past year change in program use**												
**Past year, days used the program**												
Binary variable, higher use (15+ days) vs. lower use (1-<15 days)	**1.215**	**1.048**	**1.410**	**0.010**	0.765	0.447	0.887	0.330	1.079	0.634	1.836	0.780
**B. Exposure to bikeshare or personal bike**												
**i. Categorical exposure, change in recent bike use**												
**Recently used bike (past 30 day personal or bikeshare use)**												
No bike use at follow-up	Referent				Referent				Referent			
Bike use at baseline + follow-up	**1.223**	**0.990**	**1.512**	**0.062**	0.567	0.241	0.701	0.194	0.705	0.295	1.684	0.431
New bike use at follow-up (not baseline)	**1.239**	**1.046**	**1.469**	**0.013**	0.637	0.334	0.755	0.170	1.282	0.715	2.300	0.405

SUPPLEMENT FIGURE 2. PARTIAL PREDICTED VALUES OF WITHIN-PERSON CHANGE IN TOTAL PHYSICAL ACTIVITY MINUTES (Y-AXIS) AS A FUNCTION OF WITHIN-PERSON CHANGE IN BIKESHARE USE (X-AXIS).

Notes: The plot visualizes the nonlinear smoothed function (thin-plate spline with 2 degrees of freedom) derived from a fully adjusted generalized additive model, specified for a negative binomial distribution. On the **y-axis** is the partial predicted value for past 7 day total minutes of non-walking moderate-vigorous activity (MVPA) minutes at follow-up, conditional on MVPA minutes at baseline and other covariates from the full model. On the **x-axis** is the difference in past year days used bikeshare (follow-up minus baseline).

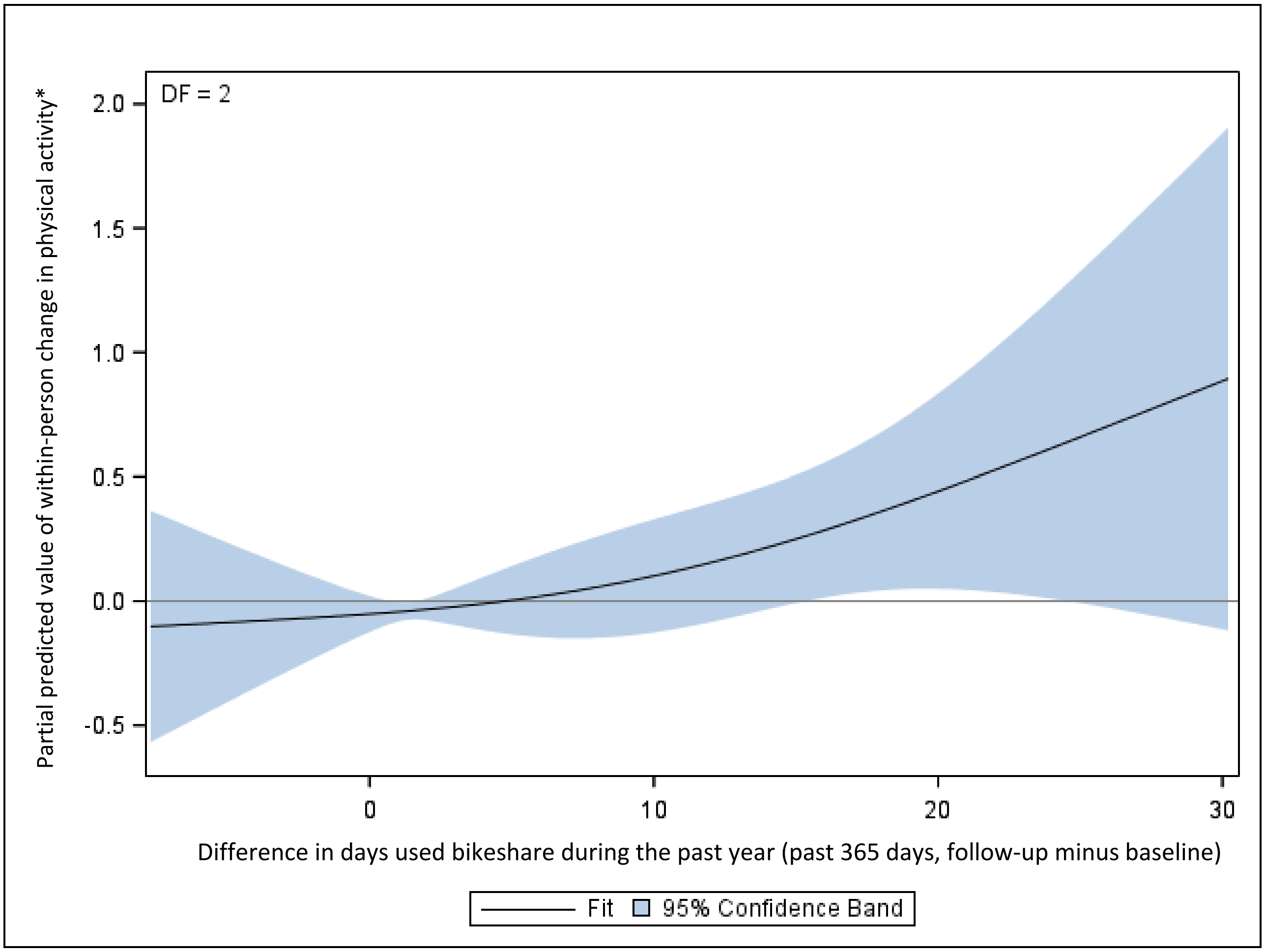

REFERENCES

1. Teitcher JEF, Bockting WO, Bauermeister JA, Hoefer CJ, Miner MH, Klitzman RL: Detecting, Preventing, and Responding to “Fraudsters” in Internet Research: Ethics and Tradeoffs. *The Journal of Law, Medicine & Ethics* 2015, 43(1):116-133.
2. Google: Google Analytics https://analytics.google.com/analytics/web/ Accessed December 1, 2017. In.; 2018.
3. U.S. Census Bureau: U.S. Census Bureau Last Accessed December 20, 2018 Available online: https://www.census.gov/quickfacts/fact/table/philadelphiacitypennsylvania,US/PST045217. In.; 2018.
4. Craig CL, Marshall AL, Sjostrom M, Bauman AE, Booth ML, Ainsworth BE, Pratt M, Ekelund U, Yngve A, Sallis JF *et al*: International physical activity questionnaire: 12-country reliability and validity. *Med Sci Sports Exerc* 2003, 35(8):1381-1395.
5. Sallis JF, Bowles HR, Bauman A, Ainsworth BE, Bull FC, Craig CL, Sjostrom M, De Bourdeaudhuij I, Lefevre J, Matsudo V *et al*: Neighborhood environments and physical activity among adults in 11 countries. *Am J Prev Med* 2009, 36(6):484-490.
6. Silsbury Z, Goldsmith R, Rushton A: Systematic review of the measurement properties of self-report physical activity questionnaires in healthy adult populations. *BMJ open* 2015, 5(9):e008430.
7. Auchincloss AH, Michael YL, Fuller D, Li S, Niamatullah S, Fillmore CE, Setubal C, Bettigole C: Design and baseline description of a cohort of bikeshare users in the city of Philadelphia. *Journal of transport & health* 2020, 16:100836.
8. Rzewnicki R, Vanden Auweele Y, De Bourdeaudhuij I: Addressing overreporting on the International Physical Activity Questionnaire (IPAQ) telephone survey with a population sample. *Public health nutrition* 2003, 6(3):299-305.
9. Tudor-Locke C, Ainsworth BE, Washington TL, Troiano R: Assigning metabolic equivalent values to the 2002 census occupational classification system. *J Phys Act Health* 2011, 8(4):581-586.
10. Ainsworth BE, Haskell WL, Herrmann SD, Meckes N, Bassett DR, Jr., Tudor-Locke C, Greer JL, Vezina J, Whitt-Glover MC, Leon AS: 2011 Compendium of Physical Activities: a second update of codes and MET values. *Med Sci Sports Exerc* 2011, 43(8):1575-1581.
11. Chetty R, Friedman J, Saez E, Turner N, Yagan D: Mobility Report Cards: The Role of Colleges in Intergenerational Mobility 1999-2013 https://opportunityinsights.org/paper/mobilityreportcards/. In: *NBER Working Paper No 23618.* National Bureau of Economic Research, Inc.; 2017.
12. Rixey RA: Station-Level Forecasting of Bikesharing Ridership:Station Network Effects in Three U.S. Systems. *Transp Res Rec* 2013, 2387(1):46-55.
13. Crowley DF, Shalaby AS, Zarei H: Access Walking Distance, Transit Use, and Transit-Oriented Development in North York City Center, Toronto, Canada. *Transp Res Rec* 2009, 2110(1):96-105.
14. El-Geneidy A, Grimsrud M, Wasfi R, Tétreault P, Surprenant-Legault J: New evidence on walking distances to transit stops: identifying redundancies and gaps using variable service areas. *Transportation* 2014, 41(1):193-210.
15. Mekuria MC, Furth PG, Nixon H: Low-stress bicycling and network connectivity https://transweb.sjsu.edu/research/Low-Stress-Bicycling-and-Network-Connectivity. In. San Jose, California: Mineta Transportation Institute, San José State University Research Foundation; 2012.
16. Hartanto K, Grigolon AB, Maarseveen MFAM, Brussel M: Developing a bikeability index in the context of transit-oriented development (TOD); 2017.
17. Ma L, Dill J: Do people's perceptions of neighborhood bikeability match "reality"? *Journal of Transport and Land Use* 2017, 10(1):291-308.
